# Benefits of mindfulness in academic settings: trait mindfulness has incremental validity over motivational factors in predicting academic affect, cognition, and behavior

**DOI:** 10.1186/s40359-022-00746-3

**Published:** 2022-03-03

**Authors:** Yuji Kuroda, Osamu Yamakawa, Masayuki Ito

**Affiliations:** 1 Center for Arts and Sciences, Fukui Prefectural University, 4-1-1 Matsuoka-Kenjojima, Eiheiji-Town, Fukui, 910-1195 Japan; 2 Faculty of Letters, Aichi Gakuin University, 12 Araike, Iwasaki-cho, Nisshin, Aichi 470-0195 Japan

**Keywords:** Achievement goals, Autonomous reasons, Controlled reasons, Implicit theory of intelligence, Mindfulness, Perceived competence

## Abstract

**Background:**

Achievement motivation research has established that motivational factors predict academic affect, cognition, and behavior. Recent studies have shown that trait mindfulness might also predict these academic outcomes. However, it remains unclear whether trait mindfulness has incremental validity over motivational factors. We hypothesized that trait mindfulness would explain unique variance in academic outcomes beyond motivational factors, because mindfulness that is characterized by the being mode of mind (i.e., a present-focused, non-striving, and accepting mind mode) would contribute to academic outcomes through unique and effective self-regulatory processes (i.e., bottom-up self-regulation of learning and present-focused, acceptance-based self-regulation of academic stress), which differ from those (i.e., top-down self-regulation of learning and future-focused, change-oriented self-regulation of academic stress) promoted by motivational factors that are characterized by the doing mode of mind (i.e., a goal-oriented, striving, and change-seeking mind mode). We tested the hypothesis by examining four established motivational factors (competence perception, implicit theory of intelligence, achievement goals, and autonomous and controlled academic reasons) and five outcome variables (test anxiety, enjoyment of learning, study strategy, mind-wandering, and help-seeking avoidance) that had been investigated in both the trait mindfulness and achievement motivation literatures.

**Methods:**

One hundred and seventy-five students (104 females) were recruited from undergraduate psychology and cultural studies classes at two universities in Japan. Trait mindfulness was assessed using the Five Facet Mindfulness Questionnaire. The other study variables were assessed using established measures as well. We conducted hierarchical multiple regression analyses to test the hypothesis.

**Results:**

Trait mindfulness predicted four of the five outcome variables (i.e., test anxiety, enjoyment of learning, mind-wandering, and help-seeking avoidance) after controlling for the motivational factors. The acting-with-awareness facet predicted three outcome variables, whereas the other facets predicted one outcome each.

**Conclusions:**

This study supports the incremental validity of trait mindfulness relative to motivational factors, suggesting that not only the doing mode of mind but also the being mode is beneficial for academic learning.

## Background

Specifying factors that predict academic outcomes is essential to understand the mechanisms of academic learning and promote student academic growth. One of the most influential factors has been identified by achievement motivation theories and research [1–3]. The theories have hypothesized that motivational factors such as achievement-related beliefs, goals, and motives have critical effects on academic learning. Empirical studies have tested this hypothesis over a half-century and found that motivational factors contribute to academic affect, cognition, and behavior [4–20].

Recent studies have begun examining another potential factor—mindfulness. While researchers originally focused on and examined the role of mindfulness in clinical settings [21, 22], they have recently extended their focus to examine its role in academic settings [23, 24]. Langer [23] and Shapiro et al. [24] argued that mindfulness has significant benefits for students’ learning and academic growth. Consistent with these views, emerging empirical studies have shown that mindfulness is associated with academic affect, cognition, and behavior [25–38], much like motivational factors.

Mindfulness is a newly introduced factor to predict academic outcomes. Thus, it is necessary to test whether it has incremental validity over motivational factors previously established in this field. However, such investigations have not been conducted thus far.

The purpose of the present study was to fill this gap by examining whether mindfulness predicts academic affect, cognition, and behavior even after controlling for motivation. In formulating the hypothesis, we focused on the differences between mindfulness and motivation in mind modes and self-regulatory mechanisms. Segal et al. [22] suggested that mindfulness is characterized by the “being” mode of mind (i.e., a present-focused, non-striving, and accepting mind mode), whereas motivation is characterized by the “doing” mode of mind (i.e., a goal-oriented, striving, and change-seeking mind mode). The doing mode inherent in motivation promotes academic outcomes through top-down, future-focused, and change-oriented self-regulation in learning situations. On the other hand, the being mode inherent in mindfulness facilitates the same academic outcomes through different self-regulatory mechanisms—bottom-up, present-focused, and acceptance-based self-regulation in learning situations. Given the differences in self-regulatory mechanisms, we hypothesized that mindfulness has a unique predictive power for academic outcomes beyond motivation. We tested the hypothesis by examining trait mindfulness, four established motivation variables (competence perception, implicit theory of intelligence, achievement goals, and academic reasons), and five outcome variables (test anxiety, enjoyment of learning, study strategy, mind-wandering, and help-seeking avoidance).

### Theory and research on achievement motivation factors

Motivation refers to the processes that instigate and sustain goal-directed activities [39]. Goals are a central component of motivation and guide and direct one’s affect, cognition, and behavior [40]. Achievement motivation refers to motivation in situations concerning individuals’ competence [2], and involves striving to develop (or acquire) and validate (or judge) competence [1].

Achievement motivation factors have been conceptualized as a trait-like individual difference variable that is relatively general and stable across achievement situations and have been hypothesized to influence affect, cognition, and behavior in various achievement situations [40, 41]. The major factors include competence self-perception (Harter’s [4] and Marsh’s theories [8]), implicit theory of intelligence (Dweck’s theory [40]), achievement goals (e.g., Elliot’s 2 × 2 achievement goal model [42]), and regulatory styles (or academic reasons; Deci and Ryan’s self-determination theory [19]). We focused on these four factors in this study for two reasons. First, these factors are necessary to capture critical motivators among students in more comprehensive ways. Students’ competence perceptions and implicit theory of intelligence involve *what they can do* and *how they can do it*, while achievement goals and academic reasons reflect *what students want to do* and *why they want to do it* [2]. Because each factor serves as a *critical but different* motivator for students, we can comprehensively capture students’ motivation by using these four constructs. Second, it was necessary to keep predictors’ levels the same in terms of generality and stability. Because we examined trait mindfulness as discussed below, we focused on trait-like motivational factors. We did not focus on such motivational factors as task value and self-efficacy because they are task- and situation-specific [5, 6, 8] and are also influenced by trait-like motivational factors [5].

Harter’s and Marsh’s theories posit that perceived academic competence plays a central role in achievement motivation [4, 7, 8]. One’s perception of academic competence is shaped by their past experiences, such as feedback from socializing agents and academic successes and failures [4, 8]. Thus, the perception of academic competence is general across academic situations and stable over time [6, 8, 43], which conceptually differs from self-efficacy that is task-specific and malleable [6, 8]. Higher perceptions of academic competence are posited to lead to adaptive academic affect, cognition, and behavior.

Dweck’s theory hypothesizes that implicit theories of intelligence influence academic outcomes [11, 40]. These implicit theories reflect individuals’ beliefs concerning the nature of intellectual ability, and include the entity theory of intelligence (i.e., the belief that intelligence is fixed and cannot be changed through effort) and the incremental theory of intelligence (i.e., the belief that intelligence is malleable and can be changed through effort). Dweck’s theory hypothesizes that the incremental theory of intelligence predicts adaptive affect, cognition, and behavior, whereas the entity theory predicts maladaptive affect, cognition, and behavior.

Goal theories posit that achievement goals affect academic outcomes. Achievement goals are defined as individuals’ purposes for engaging in competence-relevant behaviors [16]. In their 2 × 2 achievement goal model, Elliot and McGregor [42] classified achievement goals into four types according to the mastery–performance and approach–avoidance dimensions: mastery-approach goals (i.e., focus on task mastery and competence development), mastery-avoidance goals (i.e., focus on the avoidance of the failure to master tasks and to develop competence), performance-approach goals (i.e., focus on the attainment of competence relative to others), and performance-avoidance goals (i.e., focus on the avoidance of incompetence relative to others). The 2 × 2 model posits that mastery-approach goals predict adaptive affect, cognition, and behavior, whereas performance-avoidance goals predict maladaptive affect, cognition, and behavior [16, 42]. Mastery-avoidance and performance-approach goals have both adaptive and maladaptive effects, as these goals comprise both positive (i.e., mastery or approach) and negative (i.e., performance or avoidance) components [16, 42].

Self-determination theory (SDT) hypothesizes that regulatory styles play a key role in human motivation [19]. SDT posits five types of regulation, which differ according to the degree of autonomy one has in pursuing activities. From lower to higher levels of autonomy, the five types are external regulation (i.e., engaging in activities because of externally pressuring demands, such as punishment and reward), introjected regulation (i.e., engaging in activities because of internally pressuring feelings, such as guilt and shame), identified regulation (i.e., engaging in activities because of self-valued goals), integrated regulation (i.e., engaging in activities because it is congruent with one’s core interests and values), and intrinsic regulation (i.e., engaging in activities for their own sake). Individuals’ regulatory style can be specified by exploring their reasons for engaging in activities [44]. Most empirical studies have used the Self-Regulation Questionnaire (SRQ) [44] to assess the reasons [45]. The SRQ assesses four types of reasons, except for integrated reasons which are difficult to assess and empirically distinguish from identified and intrinsic reasons [19, 20, 45]. The external and introjected reasons reflect controlled reasons, whereas the identified and intrinsic reasons reflect autonomous reasons. SDT posits that individuals have innate needs for autonomy, competence, and relatedness, and that their fulfillment facilitates the development of autonomous reasons, whereas their thwarting contributes to the development of controlled reasons. SDT predicts that autonomous academic reasons lead to adaptive academic affect, cognition, and behavior, whereas controlled academic reasons lead to maladaptive affect, cognition, and behavior.

To test the above four theoretical predictions, empirical studies have used various indexes of academic affect, cognition, and behavior. Frequently used indexes include enjoyment of learning and test anxiety (affect), effective study strategies and task absorption versus distraction (cognition), and help-seeking and persistence (behavior). Previous studies using these indexes have provided evidence supporting the theoretical predictions concerning competence self-perception [4–9], implicit theory of intelligence [10–12], achievement goals [13–16], and academic reasons [17–20].

### Mindfulness and academic affect, cognition, and behavior

Mindfulness is commonly defined as ‘paying attention in a particular way: on purpose, in the present moment, and nonjudgmentally’ [21]. When defined in this way, mindfulness has two components: the regulation of attention to present-moment experience and the accepting and open orientation to present-moment experience [46]. Mindfulness can be viewed as a trait, state, or skill that can be developed through practice [47]. Trait mindfulness refers to a mindful tendency that is stable over time and general across situations and reflects between-individual differences in mindfulness [28, 32]. State mindfulness refers to a mindful state that occurs in the present moment and within a specific situation and reflects within-individual differences in mindfulness [28, 32]. In mindfulness interventions, mindfulness is viewed as a set of skills that can be learned and practiced [47]. Previous studies have found that mindfulness interventions enhance trait mindfulness measured through self-report questionnaires [48, 49], suggesting that trait mindfulness reflects a set of learned skills, but not an innate tendency.

In this study, we focused on trait mindfulness for two reasons. The first reason concerns the trend in the literature concerning mindfulness in academic settings. In evaluating the incremental validity of mindfulness over motivation, it is necessary to use outcome variables that *both* mindfulness and motivation have been found to predict. Upon reviewing the literature, most studies have focused on trait mindfulness and found that it predicts the same academic outcomes as those predicted by motivational factors (see the literature review below). In contrast, relatively few studies (except for Charoensukmongkol [27] and Senker et al. [32]) have investigated relationships between state mindfulness and academic affect, cognition, and behavior. Given the existing literature, we focused on trait mindfulness. The second reason involves the theoretical framework of the current study. As discussed later in detail, we postulate self-regulatory functioning as a mechanism through which mindfulness leads to academic outcomes. Previous studies have suggested that trait mindfulness serves as an underlying factor in the self-regulatory mechanisms [28, 31, 50]. Recent studies show that trait (i.e., situation-general) mindfulness enhances state (i.e., situation-specific) mindfulness in more situations, thereby leading individuals to self-regulate more successfully across various situations [32]. Given its wide impact on self-regulation in various situations, we focused on trait mindfulness.

Empirical studies have shown that trait mindfulness is associated with academic affect, cognition, and behavior, much like the aforementioned four motivational factors. With regard to affect, trait mindfulness was negatively associated with test and performance anxiety [25–27] and positively associated with enjoyment and intrinsic interest [28–30]; see also Howell and Buro [31] for a study using a total index of academic emotions including test anxiety and enjoyment and Senker et al. [32] for a study using indices of negative and positive academic emotions). As for cognition, trait mindfulness was negatively associated with mind-wandering [33, 34] and positively associated with absorption [35] and cognitive and metacognitive study strategies [36; see also Howell and Buro [31] for an exception). As for the behavioral variables, trait mindfulness was positively associated with help-seeking [31] and persistence [37] and negatively associated with counterproductive academic behavior [38]. All of these studies examined college student samples.

### Comparison between motivational factors and mindfulness

Two important differences exist between motivational factors and mindfulness. First, motivational factors and mindfulness show a clear difference in the modes of mind—the doing mode and the being mode [22]. The doing mode is characterized by goal strivings, change-seeking attitudes, and a focus more on the future or past than on the present. In this mode, individuals strive for particular goals and reasons, try to change internal and external states, and tend to evaluate present states in comparison with future goals or past states. In contrast, the being mode is characterized by non-striving, accepting attitudes, and a focus on present-moment experience. In this mode, people focus on being present in the here and now rather than striving for future goals or thinking about past events. They are fully attentive to and aware of present-moment events and accept them as they are, rather than trying to change and control them. The doing mode of mind is inherent in motivation and motivational factors (see their definitions), whereas the being mode is inherent in mindfulness [22].

Second, a difference exists in mechanisms through which motivational factors and mindfulness contribute to academic outcomes. Regarding the mechanisms, previous studies have suggested that both promote self-regulatory processes, which lead to academic outcomes [51, 52]. More specifically, motivational factors and mindfulness promote self-regulation of learning [31, 52] and self-regulation of academic stress [24, 40, 53]. However, we suggest that each promotes different forms of self-regulation owing to the different modes of mind.

With regard to self-regulation of learning, learners regulate their learning processes in two different modes—top-down (or goal-driven) mode and bottom-up (or data-driven) mode [54]. Competence beliefs, achievement goals, and academic motives involve the top-down mode of self-regulation [54], whereas mindfulness involves the bottom-up mode of self-regulation (see Brown et al. [50] p. 216). Top-down self-regulation indicates that learners’ personal dispositions, such as motivational tendencies that are examined in this study, drive and guide their learning processes in a particular direction, and it works mainly before and after task processing. For example, the types of goals and motives that students have for learning determine how much time and effort they spend on a given task (i.e., task-involvement) and how they deal with it (i.e., task-analysis, planning, and goal-setting before task processing). On the other hand, bottom-up self-regulation reflects that learners modulate their responses based on the current data and information during task processing. In bottom-up self-regulation, learners need to monitor ongoing task processing and to regulate negative affect such as anxiety and irritation occurring during task processing. We suggest that the doing mode of mind characterizing motivational tendencies would facilitate top-down self-regulation such as task-analysis, planning, goal-setting, and task-involvement, because this mode is goal-oriented, striving, and change-seeking in nature. On the other hand, the being mode of mind characterizing mindfulness would facilitate bottom-up self-regulation by enhancing receptivity to input and output information, facilitating open awareness of ongoing task processing, and reducing preoccupation with negative affect during task processing, as this mode is present-focused, non-striving, and accepting in nature.

Regarding self-regulation of academic stress, motivational factors characterized by the doing mode would facilitate future-focused, change-oriented coping with stress. For example, individuals who have positive motivational beliefs, goals, and reasons interpret academic difficulties and failures as good opportunities for improving their competence and continue to strive to achieve their goals and future success [40, 53]. On the other hand, mindfulness characterized by the being mode would facilitate present-focused, acceptance-based coping with academic stress [26, 50, 55]. For example, mindful individuals do not judge academic difficulties and failures as good or bad, but perceive and accept them as they are. This non-judgmental and accepting attention and awareness lead individuals to understand academic stress in a more objective and accurate way, and consequently allow them to cope with the stress effectively [26, 50, 55].

In sum, mindfulness and motivation fundamentally differ in the modes of mind, and thus, facilitate different forms of self-regulation. While these differences exist, empirical studies have shown that trait mindfulness is positively correlated with competence self-perceptions [28], incremental intelligence beliefs [31], mastery-approach goals [31], and autonomous reasons [30], suggesting that mindful tendencies relate to positive motivational tendencies. These correlations are reasonable because mindful tendencies and positive motivational tendencies both serve adaptive functions and thus are likely to coexist (and be successfully coordinated) in healthy individuals. However, given the correlations, it is possible that the effects of trait mindfulness on academic outcomes might overlap with (and thus be attributed to) the effects of motivational factors.

### Purpose and hypothesis of the present study

Motivational factors are established predictors of academic outcomes and involve the doing mode of mind which promotes top-down, future-focused, and change-oriented self-regulation. Trait mindfulness is a newly introduced construct to predict academic outcomes and reflects the being mode of mind which promotes bottom-up, present-focused, and acceptance-based self-regulation. While trait mindfulness and motivational factors differ conceptually and functionally, they are correlated because both serve adaptive functions and likely coexist in healthy individuals. Given that trait mindfulness is a newly introduced construct and is also correlated with motivational factors previously established in this field, it is necessary to investigate whether trait mindfulness uniquely predicts academic outcomes after controlling for motivational factors (i.e., whether trait mindfulness has incremental validity over motivational factors). This investigation allows us to evaluate the pure effect of the being mode inherent in mindfulness as it partials out the effects of doing-mode variables (motivational factors). However, such an investigation has not been conducted so far.

The purpose of the present study was to fill this significant gap in the literature. In formulating the hypothesis, we focused on the differences between mindfulness and motivation in mind modes and self-regulatory mechanisms as discussed above. The being mode inherent in mindfulness would contribute to academic outcomes by facilitating unique and effective self-regulatory processes, which differ from those facilitated by the doing mode inherent in motivational factors. Given the differences, we hypothesized that trait mindfulness would uniquely predict academic affect, cognition, and behavior after controlling for motivational factors.

To test the hypothesis, it was necessary to use outcome variables that had been examined in both the trait mindfulness and achievement motivation literatures. Given the aforementioned empirical findings in both literatures, we examined test anxiety and enjoyment of learning (affective variables), study strategy and distraction (mind-wandering) (cognitive variables), and help-seeking avoidance (behavioral variable). Regarding the study sample, previous studies concerning achievement motivation have examined students ranging from elementary school to college, while studies on trait mindfulness and academic affect, cognition, and behavior have focused on college students, as discussed above. Considering existing research on trait mindfulness, we examined college students.

## Methods

### Participants

The study participants were recruited from undergraduate psychology and cultural studies classes at two universities in Japan. Initially, 179 students agreed to participate in the investigation and answered the questionnaires. Of these, four students were excluded due to incomplete data for both mindfulness and motivation (predictors) or sex (a controlling variable). The remaining 175 students (104 females and 71 males; *M*_age_ = 19.52 years, *SD* = 1.51) were examined in the subsequent analyses.

### Measures

#### Mindfulness

We used the Japanese-translated version [56] of the Five Facet Mindfulness Questionnaire (FFMQ) [47]. The FFMQ measures the general tendency to be mindful in daily life and comprises five subscales reflecting the essential components of mindfulness: observing, describing, acting with awareness, nonreactivity to inner experiences, and nonjudging of inner experiences. The observing subscale measures the ability to notice or attend to internal and external stimuli (eight items; e.g., “I pay attention to sounds, such as clocks ticking, birds chirping, or cars passing”). The describing subscale assesses the ability to verbally describe one’s thoughts and feelings in the present moment (eight items; e.g., “I can usually describe how I feel at the moment in considerable detail”). The acting-with-awareness subscale measures the tendency to be attentive to and aware of present-moment activities, as opposed to acting automatically without paying attention (eight items; e.g., “I do jobs or tasks automatically, without being aware of what I’m doing” [reverse-scored]). The nonreactivity subscale evaluates the tendency to allow thoughts and feelings to come and go without getting caught up in them (seven items; e.g., “Usually when I have distressing thoughts or images, I just notice them and let them go”). Finally, the nonjudging subscale measures the individual’s tendency to adopt a non-evaluative, accepting stance toward thoughts and feelings as opposed to judging them as good or bad (eight items; e.g., “I make judgments about whether my thoughts are good or bad” [reverse-scored]). Participants rated each item on a 5-point Likert scale ranging from 1 (*never or very rarely true*) to 5 (*very often or always true*). Higher scores indicate a higher level of each facet of mindfulness. Studies [47, 56] confirmed the factorial and construct validity of this measure and the internal consistency of each subscale.

#### Competence self-perception

We used the intellectual ability subscale of the Japanese-translated version of the Self-Perception Profile for College Students (SPPCS-JV; Maeshiro et al. [57], original SPPCS by Neemann and Harter [43]) to measure competence self-perception. This subscale assesses students’ perception of their general intellectual competence (four items; e.g., “I feel I am intelligent”) and has construct validity and internal consistency [43, 57]. The SPPCS-JV utilizes the revised question format by Wichstrøm [58], which asks respondents to rate each item on a 4-point Likert scale ranging from 1 (*describes me very poorly*) to 4 (*describes me very well*). A higher score indicates higher perceived competence.

#### Implicit theory of intelligence

We employed the Japanese-translated version [59] of the Implicit Theory of Intelligence Scale [60]. This scale has construct validity and internal consistency [59, 60] and comprises three items (e.g., “You have a certain amount of intelligence and you really can’t do much to change it”). Participants rated each item on a 6-point Likert scale ranging from 1 (*strongly disagree*) to 6 (*strongly agree*), with a higher score indicating a stronger belief that intelligence is fixed and unchangeable; conversely, the lower respondents’ scores are, the stronger is their belief that intelligence is malleable and changeable [60].

#### Achievement goals

We used the Japanese-translated version [61] of the Achievement Goal Questionnaire-Revised [62]. This questionnaire contains four subscales, each consisting of three items: mastery-approach goals (e.g., “My aim is to completely master the material presented in classes”), mastery-avoidance goals (e.g., “My goal is to avoid learning less than it is possible to learn”), performance-approach goals (e.g., “My goal is to perform better than the other students”), and performance-avoidance goals (e.g., “My aim is to avoid doing worse than the other students”). Participants rated each item on a 5-point Likert scale ranging from 1 (*strongly disagree*) to 5 (*strongly agree*). Higher scores indicate a higher level of each goal. Previous studies [61–63] showed the factorial and construct validity of this questionnaire and the internal consistency of each subscale.

#### Academic reasons

We employed the Japanese short version [64] of the SRQ [44]. This questionnaire comprises four subscales assessing individuals’ intrinsic, identified, introjected, and external reasons for studying. Nishimura et al. [64] confirmed the factorial and construct validity of this measure and the internal consistency and test–retest reliability of each subscale. Each subscale consists of five items, to which participants respond on a 4-point scale ranging from 1 (*not at all true*) to 4 (*very true*). Sample items include “Because solving problems is interesting” (intrinsic reason), “Because I want to realize my dream” (identified reason), “Because I feel ashamed if I don’t do well in my studies” (introjected reason), and “Because others yell at me if I don’t do it” (external reason). One item from the identified reason subscale (“Because I want to go to the high school and college that I wish to go to”) was modified for college students (“Because I want to get a job that I wish for”). Based on previous studies [65], we created composite scores for autonomous and controlled reason by adding intrinsic and identified reason scores and introjected and external reason scores, respectively. Higher scores indicate higher levels of autonomous and controlled reasons.

#### Test anxiety

We used the test anxiety subscale of the Japanese-translated version [66] of the Achievement Emotions Questionnaire [67]. This subscale measures anxiety before and during tests or exams (twelve items; e.g., “Before the exam I feel nervous and uneasy”) and has construct validity and internal consistency [66, 67]. Participants rated each item on a 5-point Likert scale ranging from 1 (*strongly disagree*) to 5 (*strongly agree*), with a higher score indicating higher test anxiety.

#### Enjoyment

To measure students’ enjoyment and fun in studying, we used the Intrinsic Interest Scale [68]. It is based on Elliot and Church’s scale [69], and consists of five items (e.g., “I think studying is fun”). A higher score indicates greater enjoyment. Tanaka and Yamauchi [68] showed the construct validity and internal consistency of this scale.

#### Study strategies

We used the Japanese-translated version [70] of the lack-of-strategy subscale of the Study Strategy Scale [71]. This subscale assesses the lack of an organized approach to studying and has construct validity and internal consistency (three items; e.g., “I often find that I don’t know what to study or where to start”). Participants rated each item on a 5-point Likert scale ranging from 1 (*not at all true of me*) to 5 (*very true of me*), and higher scores indicate a higher level of lack of study strategies.

#### Mind-wandering

We employed the Japanese-translated version [72] of the Mind-Wandering Questionnaire [73]. This questionnaire assesses the frequency of the interruption of task-focus (distraction) by task-unrelated thought (five items; e.g., “I mind-wander during lectures or presentations”) and has construct validity and internal consistency [72, 73]. Participants rated each item on a 6-point Likert scale ranging from 1 (*almost never*) to 6 (*almost always*). Higher scores indicate greater levels of mind-wandering.

#### Help-seeking avoidance

We employed the help-seeking avoidance subscale of the Academic Help Seeking Scale [74], which is based on Ryan and Pintrich’s scale of help-seeking avoidance [75]. This subscale evaluates whether individuals avoid help-seeking in academic contexts when needed (three items; e.g., “When I face a task that is hard to solve on my own, I do not ask teachers or friends and give up solving it”) and has construct validity and internal consistency [74]. Participants rated each item on a 5-point Likert scale ranging from 1 (*not at all true of me*) to 5 (*very true of me*). A higher score indicates greater help-seeking avoidance.

### Procedure

This study was approved by the ethics committee of the university with which the first and second authors were affiliated. The authors administered the questionnaires to the participants in the classroom. All participants were assured that their answers would be kept anonymous and confidential, that their participation was voluntary, and that they could withdraw from the study at any time without penalty. After they provided their written consent to participate, they answered the questionnaires.

### Data analysis

We conducted hierarchical multiple regression analyses to test the study hypothesis. In the first step, sex was entered as a control variable. In the second step, the four motivational factors were added. Finally, in the third step, trait mindfulness was included. If the increment in *R*^*2*^ at the third step was significant, then trait mindfulness explained unique variance in the outcome variables beyond the motivational factors—that is, it was deemed to have incremental validity in predicting the outcome variables. The outcome variables were academic affect, cognition, and behavior (five indexes). IBM SPSS Statistics 19.0 (IBM Corp., Armonk, NY) was used for the analyses.

## Results

### Descriptive statistics

Table 1 shows the mean, standard deviation, and Cronbach’s alpha coefficient of each measure. The alpha coefficients ranged from 0.64 to 0.91, indicating acceptable to good internal consistency of each measure. Table 1 also indicates the Pearson’s correlation coefficients among the study measures. In line with the hypothesis, acting with awareness, describing, and nonjudging were significantly correlated with four outcome variables, and nonreactivity was significantly correlated with two outcome variables. Observing showed an unexpected significant positive correlation with mind-wandering. This finding accords with those of several previous studies [47, 56] showing that observing had significant positive relationships with maladaptive variables. The four motivational factors showed the expected significant correlations with outcome variables, with a few exceptions.

**Table 1:** Means, Standard Deviations, and Reliability of Each Measure and Correlations Between Measures

		*N*	*M*	SD	1	2	3	4	5	6	7	8	9
1	Mindfulness: observing	173	22.61	4.68	.64								
2	Mindfulness: describing	175	22.63	6.06	.03	.87							
3	Mindfulness: act-with-awareness	169	25.24	5.44	− .20**	.33**	.82						
4	Mindfulness: nonreactivity	173	19.91	3.90	.07	.28**	.13	.67					
5	Mindfulness: nonjudging	174	23.23	5.26	− .35**	.17*	.30**	.15	.78				
6	Competence perception	175	8.58	2.25	.01	.30**	.23**	.31**	.21**	.66			
7	Implicit theory of intelligence	174	9.06	3.21	− .02	− .24**	− .30**	− .06	− .22**	− .13	.85		
8	Mastery-approach goals	175	10.82	2.39	.02	.01	.13	− .06	− .06	.08	− .12	.75	
9	Mastery-avoidance goals	175	9.34	2.55	.14	.07	− .02	.03	− .07	.22**	.03	.48**	.85
10	Performance-approach goals	174	9.07	3.16	.10	− .05	− .01	.07	− .11	.18*	.00	.53**	.28**
11	Performance-avoidance goals	175	9.28	3.18	.10	− .05	− .10	.00	− .17*	.05	.10	.27**	.27**
12	Autonomous academic reasons	173	27.45	5.40	.11	.11	.18*	.17*	.06	.22**	− .20**	.40**	.26*
13	Controlled academic reasons	172	22.60	5.10	.08	− .17*	− .07	.04	− .10	.03	.19*	.23**	.16*
14	Test anxiety	175	34.97	10.12	.08	− .20**	− .27**	− .14	− .16*	− .09	.16*	.13	.12
15	Enjoyment	173	16.21	5.40	.10	.04	.21**	.07	.14	.19*	− .14	.35**	.29**
16	Lack of study strategies	175	10.06	3.05	.13	− .24**	− .14	− .19*	− .24*	− .35**	.02	− .05	− .14
17	Mind-wandering	173	18.65	4.15	.16*	− .32**	− .57**	− .24**	− .28**	− .28**	.21**	− .17*	− .07
18	Help-seeking avoidance	174	7.37	3.15	.03	− .34**	− .31**	− .10	− .19*	− .16*	.16*	− .18*	− .16*
19	Sex	175	0.59	0.49	.09	− .20**	.07	− .15	.02	− .13	− .02	.07	.01

		*N*	*M*	*SD*	10	11	12	13	14	15	16	17	18
1	Mindfulness: observing	173	22.61	4.68									
2	Mindfulness: describing	175	22.63	6.06									
3	Mindfulness: act-with-awareness	169	25.24	5.44									
4	Mindfulness: nonreactivity	173	19.91	3.90									
5	Mindfulness: nonjudging	174	23.23	5.26									
6	Competence perception	175	8.58	2.25									
7	Implicit theory of intelligence	174	9.06	3.21									
8	Mastery-approach goals	175	10.82	2.39									
9	Mastery-avoidance goals	175	9.34	2.55									
10	Performance-approach goals	174	9.07	3.16	.88								
11	Performance-avoidance goals	175	9.28	3.18	.63**	.90							
12	Autonomous academic reasons	173	27.45	5.40	.36**	.16*	.86						
13	Controlled academic reasons	172	22.60	5.10	.52*	.50*	.15*	.77					
14	Test anxiety	175	34.97	10.12	.19*	.34**	.06	.25**	.89				
15	Enjoyment	173	16.21	5.40	.24**	.10	.70**	.12	− .07	.91			
16	Lack of study strategies	175	10.06	3.05	− .03	.10	− .25**	.17*	.32**	− .25**	.88		
17	Mind-wandering	173	18.65	4.15	− .08	.02	− .29**	.03	.23**	− .29**	.33**	.72	
18	Help-seeking avoidance	174	7.37	3.15	− .10	.07	− .38**	.09	.14	− .32**	.34**	.43**	.89
19	Sex	175	0.59	0.49	.05	− .06	.05	.06	− .01	.04	.14	− .03	− .04

*p < .05, **p < .01 (two-tailed tests). Sex was coded as 0 for males and 1 for females. Values in the diagonal are Cronbach's alpha coefficients. *N* ranges from 169 to 175 due to missing data (response rate = 94% to 98%)

Competence perception, implicit theory of intelligence, and academic reasons showed significant correlations with three to four facets of trait mindfulness. Achievement goals were not significantly correlated with the facets of mindfulness, except for a significant negative correlation between performance avoidance goals and nonjudging.

Sex showed a significant correlation with describing and a marginally significant correlation with lack of study strategies (*p* = 0.061). Thus, we entered sex as a covariate in the first step of hierarchical regression analyses.

### Test of hypothesis

Table 2 shows the results of hierarchical regression analyses. Motivational factors explained a significant proportion of the variance in all five outcome variables (see *R*^*2*^ in Step 2). An examination of the relative effect of each factor revealed that autonomous academic reason was a significant predictor of four outcome variables. Competence perception significantly predicted two outcome variables, while performance avoidance goal and controlled academic reason significantly predicted one outcome each. Adding the subscale scores of trait mindfulness in the third step yielded significant increments in *R*^*2*^ (*ΔR*^*2*^) for four of the five outcome variables (i.e., test anxiety, enjoyment, mind-wandering, and help-seeking avoidance). An examination of the relative effect of each subscale revealed that acting with awareness was a significant predictor of three outcome variables (i.e., test anxiety, enjoyment, and mind-wandering), while describing, nonreactivity, and nonjudging significantly predicted one outcome each (help-seeking avoidance, mind-wandering, and enjoyment, respectively). Observing was a marginally significant predictor of enjoyment (*p* = 0.059). Overall, the results support the hypothesis.

**Table 2:** Hierarchical multiple regression analyses predicting the five outcome variables from motivational factors and mindfulness

Predictor variables	Outcome variables									
	Test anxiety (*N* = 161)		Enjoyment (*N* = 160)		Lack of study strategies (*N* = 161)		Mind-wandering (*N* = 159)		Help-seeking avoidance (*N* = 160)	
	*ΔR*^*2*^	*β*	*ΔR*^*2*^	*β*	*ΔR*^*2*^	*β*	*ΔR*^*2*^	*β*	*ΔR*^*2*^	*β*
Step 1	.00		.00		.03*		.00		.00	
Sex		.02		.01		.17*		.01		.01
Step 2	.16**		.52**		.24**		.15**		.18**	
Sex		.01		− .01		.11		− .01		.01
Competence perception		− .09		.01		− .30**		− .23**		− .08
Implicit theory of intelligence		.12		.00		− .12		.10		.05
Mastery-approach goals		.04		.08		.07		− .15		.00
Mastery-avoidance goals		.02		.09		− .04		.09		− .06
Performance-approach goals		− .11		− .10		− .08		.07		− .06
Performance-avoidance goals		.33**		.00		.06		.01		.11
Autonomous academic reasons		.06		.68**		− .27**		− .20*		− .35**
Controlled academic reasons		.11		.03		.27**		.00		.13
Step 3	.07*		.04*		.02		.26**		.11**	
Sex		.00		− .07		.09		− .02		− .01
Competence perception		.02		− .02		− .26**		− .03		.04
Implicit theory of intelligence		.04		.05		− .13		− .04		− .06
Mastery-approach goals		.07		.05		.08		− .10		.03
Mastery-avoidance goals		− .01		.13^†^		− .07		.00		− .08
Performance-approach goals		− .15		− .07		− .10		.00		− .13
Performance-avoidance goals		.33**		.00		.06		− .01		.13
Autonomous academic reasons		.10		.66**		− .27**		− .13		− .32**
Controlled academic reasons		.10		.02		.26**		.01		.09
Mindfulness: observing		− .01		.12^†^		.12		.08		.01
Mindfulness: describing		− .11		− .10		− .08		− .10		− .29**
Mindfulness: acting-with-awareness		− .21*		.13*		.01		− .44**		− .14
Mindfulness: nonreactivity		− .07		− .07		− .02		− .14*		.06
Mindfulness: nonjudging		− .04		.17**		− .05		− .09		− .09

^†^*p* < .10, * *p* < .05, ** *p* < .01 (two-tailed tests). *ΔR*^2^ = increment in *R*^2^. Sex was coded as 0 for males and 1 for females. *N* ranges from 159 to 161 due to missing data (response rate = 89% to 90%)

## Discussion

Consistent with our hypothesis, trait mindfulness predicted four of the five academic outcomes after controlling for motivational factors. This result means that the unique characteristics of mindfulness that do not exist in motivational factors predicted academic outcomes. The mindfulness-specific characteristics involve the being mode of mind (i.e., a present-focused, non-striving, and accepting mind mode), as opposed to the doing mode of mind (i.e., a goal-oriented, striving, and change-seeking mind mode) that characterizes motivational factors. Why did the being mode of mind predict academic outcomes beyond the doing mode? We suggest that the being mode would facilitate two unique and effective self-regulatory processes—bottom-up self-regulation of learning and present-focused, acceptance-based self-regulation of academic stress—that differ from those facilitated by the doing mode (see Introduction for details). We discuss our findings in light of these self-regulatory processes.

### Mindfulness and self-regulatory processes

Acting with awareness showed a positive association with enjoyment of learning and negative associations with test anxiety and mind-wandering. Acting with awareness refers to the tendency to be attentive to and aware of present-moment experience, which reflects a central element of mindfulness and the being mode of mind. This facet promotes bottom-up self-regulation of learning. Specifically, it serves to focus learners’ attention on moment-by-moment input and output information during task processing and to enhance their awareness of moment-by-moment task processing. This self-regulatory process would facilitate engagement in a task, thereby preventing mind-wandering and enhancing enjoyment of learning. Acting with awareness would also promote present-focused self-regulation of academic stress. It leads students to focus their attention on present (but not past and future) and real (but not recalled and imaginary) experiences after they have experienced academic stress and when they possibly experience academic stress in the future. By focusing on present and real events, students are less likely to ruminate about past and future negative academic events [22] and are more likely to use coping strategies flexibly and effectively [26]. These self-regulatory processes would serve to reduce academic stress including test anxiety.

Nonjudging and observing predicted enjoyment of learning. Nonjudging refers to the tendency to adopt a non-evaluative, accepting stance toward thoughts and feelings, which is a critical component of mindfulness and the being mode of mind. Observing refers to the ability to notice or attend to internal and external stimuli, which is a prerequisite for mindful states and the being mode. These two facets (i.e., non-judgmental and accepting observation) would facilitate bottom-up self-regulated learning in ways that lead to enjoyment of learning. Specifically, non-judgmental and accepting observation would enhance receptivity to input and output information and open awareness of ongoing learning activities, which would allow learners to notice important or interesting information in a task and lecture that is likely to be overlooked by a fixed perspective (see also Grund et al. [29] for a related discussion) and important gaps between existing knowledge and real phenomena. These intellectual experiences during bottom-up self-regulated learning would enhance students’ enjoyment of learning.

Nonreactivity showed a negative association with mind-wandering (i.e., distraction by task-unrelated thoughts). Nonreactivity reflects the ability to allow unwanted thoughts and feelings to come and go without reacting to them, which involves an accepting and non-striving attitude (i.e., not trying to control and change undesirable states but allowing and accepting them). This ability would help learners successfully regulate and reduce negative affect such as irritation, anger, and anxiety that is likely to surface during bottom-up self-regulated learning [54]. Successful affect regulation would allow learners to concentrate more on a task. Nonreactivity would also help learners regulate task-unrelated thoughts occurring during bottom-up self-regulated learning. Based on ironic process theory [76] that proposes the paradoxical effects of thought suppression, trying harder to suppress a task-unrelated thought ironically leads to more preoccupation with that thought. In contrast to thought suppression, nonreactivity serves not to try to control task-unrelated thoughts but to let them go. This function would reduce preoccupation with task-unrelated thoughts, and consequently protect against distraction.

Describing showed a negative association with help-seeking avoidance in the face of academic difficulties. We suggest that describing ability may be related to social interaction skills such as verbal expression skills, which may reduce help-seeking avoidance. Describing reflects one’s ability to describe what and how they are thinking and feeling in the present moment. When facing academic difficulties, students with this ability will be able to successfully verbalize and express their feelings and thoughts concerning difficulties. These verbal expression skills may lead the students to seek help willingly. Consistent with these views, previous studies suggest that describing would facilitate communication, thereby enabling receipt of social support and promoting problem-solving [77].

### Relationships among mindfulness, motivation, and academic outcomes

We found that no facets of trait mindfulness predicted study strategy after controlling for motivational factors. At the correlational level, describing, nonreactivity, and nonjudging were significantly associated with study strategy. However, they were also significantly correlated with competence perception that predicted study strategy. Thus, the effects of these mindfulness facets on study strategy might be attributed to the effect of competence perception. Study strategy examined in this study reflects an organized approach to study, such as planning what to study and where to start. This strategy involves task analysis and goal setting, which might be facilitated more by the doing-mode variables like motivational factors than by the being-mode variable like mindfulness.

An alternative explanation for this result is that mindfulness might predict study strategy use indirectly through the mediation of competence perception. This explanation is consistent with recent findings from the perspective of SDT [19, 28, 50, 78] (see also Howell and Buro [31] and Grund and Senker [55] for related findings). Studies have integrated mindfulness into SDT to show that having mindful tendencies is more likely to lead to satisfying needs for competence, autonomy, and relatedness [79] because mindfulness allows individuals to perceive and monitor their inner and outer states in comprehensive and receptive ways [19, 50, 55, 80]. Need satisfaction, in turn, promotes autonomous motivation, which produces adaptive academic outcomes [19]. Based on these findings, mindfulness not only predicts academic outcomes independently of adaptive motivation, but also produces outcomes indirectly by inducing adaptive motivation.

### Contributions and limitations of this study

This study contributes to the literature in three important ways. First, this study is the first to demonstrate the incremental validity of mindfulness in predicting academic outcomes. Because mindfulness is a newly introduced factor to predict academic outcomes, it is crucial to demonstrate whether it explains unique variance in academic outcomes beyond factors established in this field. Our findings support the incremental validity of trait mindfulness over motivational factors well-established in this field, which corroborates previous findings and encourages future research concerning the academic benefits of trait mindfulness. Second, this study highlights the academic benefits of the being mode of mind, as opposed to the doing mode. This argument is reasonable given that trait mindfulness predicted academic outcomes after controlling for motivational factors. While previous studies demonstrated the academic benefits of doing-mode variables like motivational factors, there is little evidence showing the potentials of the being mode in academic learning. Given the previous and present findings, both the doing and being modes are important in academic settings. Using the two modes flexibly according to the situation may be the key to successful learning. Finally, this study suggests that mindfulness and motivational factors would contribute to academic outcomes through different mechanisms. Previous studies have suggested that mindfulness (e.g., see Ostafin et al. [51] for a review) and motivational factors (e.g., see Schunk & Zimmerman [52] for a review) both facilitate effective self-regulation, thereby leading to adaptive outcomes. Regarding the specific types of self-regulation, we suggest that motivational factors characterized by the doing mode of mind would promote top-down, future-focused, and change-oriented self-regulation, whereas mindfulness characterized by the being mode of mind would facilitate bottom-up, present-focused, and acceptance-based self-regulation.

This study also has practical implications. Our findings suggest that educators might be able to promote students’ academic outcomes by cultivating their mindfulness. Meditation is a well-known practice to cultivate mindfulness and has recently begun to be introduced in educational settings [24]. In conducting meditation programs, educators might focus especially on cultivating students’ attention to and awareness of present-moment experience, because both the present study and previous studies [29, 32] showed that the acting-with-awareness facet is a promising predictor of academic outcomes. Concentrative meditation (focusing attention on a single object such as breathing) would be helpful to cultivate present-focused attention and awareness.

Several limitations concerning this study require consideration. First, because the study design was cross-sectional and correlational, we have limited ability to judge the causality of the observed relationships. Experimental or longitudinal studies are necessary to provide plausible evidence for the causal directions of the associations. The second issue concerns the generalizability of the results due to the nature of the sample. We investigated samples from Japan; thus, we need to consider potential cultural differences. Many previous studies have examined Western samples and shown that trait mindfulness produces desirable academic and achievement-related outcomes. Recent studies examining Eastern samples have yielded the same findings in the academic domains [26, 27, 34, 36] and in achievement-related domains [81–83]. Based on these findings, we speculate that among people in both cultures, trait mindfulness supports achievement-relevant processes and outcomes. However, to confirm this suggestion, further investigations in both cultures are necessary. Another issue concerning the study sample was that we used a college student sample and its size was relatively small. To confirm the generalizability of the study findings, it is necessary in future research to examine different age group and to use larger samples. Third, the current study does not provide direct evidence for our proposal that mindfulness and motivation facilitate different self-regulatory processes. The proposal is theoretically sound and is consistent with previous studies indicating that mindfulness and self-control facilitate different self-regulation processes [55], but lacks empirical evidence. Future empirical studies are necessary to verify the differences between mindful and motivated self-regulation. Fourth, because we focused on the unique contribution of trait mindfulness to academic outcomes beyond motivational factors, we did not examine whether trait or state mindfulness and motivation combine to predict academic outcomes. Mindfulness might predict academic outcomes not only directly (i.e., the unique contribution of mindfulness beyond motivational factors) but also indirectly through adaptive motivational processes [31, 55, 78, 79]. Mindfulness might also moderate or mediate the relationship between motivation and academic outcomes [80]. Therefore, research is necessary to further examine not only how mindfulness and motivation differ in ways that produce academic benefits (the unique mechanisms of mindfulness and motivation), but also how these variables combine to produce academic benefits (the joint mechanisms of mindfulness and motivation). Fifth, we had to rely on self-reported measures, which are subject to response bias; researchers should use more objective measures, such as laboratory task performance and physiological measures, in the future. Finally, this study did not cover all possible outcome variables. To provide more evidence for the incremental validity of mindfulness, it is necessary to investigate other variables than those examined in this study.

## Conclusions

We found that trait mindfulness predicted academic affect, cognition, and behavior after controlling for achievement motivation factors. The findings support the incremental validity of trait mindfulness and demonstrate the academic benefits of the being mode inherent in mindfulness. The findings also suggest that cultivating mindfulness (especially, the acting-with-awareness facet) in students might help improve their academic outcomes. Our findings have both theoretical and practical importance, which encourages future empirical studies and educational interventions.

## Data Availability

The datasets generated and analyzed during the current study are not publicly available due to ongoing analyses but are available from the corresponding author on reasonable request.

## Abbreviations

SDT: Self-determination theory
FFMQ: Five Facet Mindfulness Questionnaire
SPPCS: Self-Perception Profile for College Students
SPPCS-JV: Japanese-translated version of the SPPCS
SRQ: Self-Regulation Questionnaire
